# A novel WS_2_ nanowire-nanoflake hybrid material synthesized from WO_3_ nanowires in sulfur vapor

**DOI:** 10.1038/srep25610

**Published:** 2016-05-16

**Authors:** Georgies Alene Asres, Aron Dombovari, Teemu Sipola, Robert Puskás, Akos Kukovecz, Zoltán Kónya, Alexey Popov, Jhih-Fong Lin, Gabriela S. Lorite, Melinda Mohl, Geza Toth, Anita Lloyd Spetz, Krisztian Kordas

**Affiliations:** 1 Microelectronics and Materials Physics Laboratories, Department of Electrical Engineering, University of Oulu, P.O. Box 4500, FI-90014 Oulu, Finland; 2 Department of Applied and Environmental Chemistry, University of Szeged, Rerrich Bela ter 1, H-6720 Szeged, Hungary; 3 MTA-SZTE “Lendület” Porous Nanocomposites Research Group, Rerrich Bela ter 1, H-6720 Szeged, Hungary; 4 MTA-SZTE Reaction Kinetics and Surface Chemistry Research Group, Rerrich Bela ter 1 H-6720 Szeged, Hungary; 5 Optoelectronics and Measurement Techniques Laboratory, Department of Electrical Engineering, University of Oulu, P.O. Box 4500, FI-90014 Oulu, Finland; 6 Department of Physics, Chemistry and Biology, Linköping University, SE-58183 Linköping, Sweden

## Abstract

In this work, WS_2_ nanowire-nanoflake hybrids are synthesized by the sulfurization of hydrothermally grown WO_3_ nanowires. The influence of temperature on the formation of products is optimized to grow WS_2_ nanowires covered with nanoflakes. Current-voltage and resistance-temperature measurements carried out on random networks of the nanostructures show nonlinear characteristics and negative temperature coefficient of resistance indicating that the hybrids are of semiconducting nature. Bottom gated field effect transistor structures based on random networks of the hybrids show only minor modulation of the channel conductance upon applied gate voltage, which indicates poor electrical transport between the nanowires in the random films. On the other hand, the photo response of channel current holds promise for cost-efficient solution process fabrication of photodetector devices working in the visible spectral range.

Metal chalcogenides have opened unexpected opportunities for novel multifunctional materials with potential application in nano and optoelectronics. In particular, nanostructures of group 4–6 transition metal dichalcogenides (TMDCs) are recently getting an enormous attention due to the their two-dimensional graphene-like layered structure and semiconducting behavior^1^,^2^,^3^. The band gap of dichalcogenides shows a systematic decrease with the increasing metallic nature of the chalcogen following the series in the main group 6 elements as S, Se and Te. However, when decreasing the number of layers in TMDCs, the band gap increases, e.g. 1.4 eV (bulk) to 2.1 eV (single layer) for WS_2_. Since the band gap values of TMDCs are comparable to that of Si (1.1 eV), transistor structures with similar low voltage operation are expected. As demonstrated lately, extremely high on/off channel current ratios (e.g. 10^5^ for flakes of WS_2_^4^, 10^7^ for WS_2_ flakes sandwiched between h-BN sheets^5^, and 10^8^ for MoS_2_ monolayers^6^) can be achieved without impurity doping or any particular band gap engineering, unlike in gapless graphene. Another interesting feature of several TMDCs is the switch from indirect band-to-band transition of multi-layered materials to direct gap when dealing with single-layer sheets. Accordingly, single-layer TMDCs may show photoluminescence^7^, which is particularly interesting for novel devices in optoelectronics^8^,^9^ and sensing^10^.

Chemical^11^,^12^,^13^ and mechanical^14^,^15^ exfoliation methods are the two most commonly used techniques to produce single or few-layered dichalcogenides. Chemical exfoliation is based on an insertion of ions (typically Li^+^) in the layered material to make the adjacent sulfide layers more separated or expanded (chemical intercalation) by which the van der Waals interaction is weakened. Then a mechanical force is applied (e.g. sonication) to separate the intercalated layers that become suspended in the solvent in the form of mono or few-layered flakes^16^,^17^. Mechanical exfoliation proceeds in a similar vein as first described for graphene synthesis^18^, i.e. by peel-off from bulk or multi-layered crystals. The TMDC crystal is repeatedly cleaved using e.g. scotch tape until a very thin layer of the materials remains on the surface of the tape, from which the atomically thin films are transferred to a target substrate^19^. The synthesis of other types of dichalcogenides is described in detail elsewhere for interested readers^20^,^21^.

Both mechanical and chemical exfoliation methods are tedious, and the immobilization of individual single TMDC flakes on chips for testing or to produce devices for commercial purposes is cumbersome. Accordingly, robust methods to fabricate nanoflakes of TMDCs would help in the practical exploitation of these fascinating materials. In this work, we discuss the synthesis of WS_2_ nanowires and nanowire-nanoflake hybrid materials by a simple single-step sulfurization from hydrothermally grown WO_3_ nanowires. The geometry of the prepared nanostructure is novel, as the flakes reside directly on the surface of nanowires and are a direct continuation of the nanowire crystal structure. This direct crystallographic interconnection between two dimensional and one dimensional structures hold the possibility to exhibit new optical and electrical properties. The obtained semiconducting hybrid nanomaterials display semiconducting behavior with direct band-to-band transition suggesting applications in field-effect transistors and particularly in photodetector devices without the need for manipulating individual nanoparticles.

## Experimental

### Synthesis of WO_3_ nanowires and their conversion to WS_2_ nanohybrids

WO_3_ nanowires were synthesized by the hydrothermal method as described in our earlier work^22^. In a typical process, 18.74 g of Na_2_WO_4_·2H_2_O (Sigma-Aldrich, ACS reagent 99%) and 22.49 g of Na_2_SO_4_ (Sigma-Aldrich, ACS reagent ≥99.0%) were dissolved in 600 mL distilled water and the pH = 1.5 was adjusted using 3 M HCl solution. After 10 min stirring, the solution was transferred to an autoclave (Parr Series 4520 Bench Top Reactor with PTFE lining) and stirred (35 rpm) for 48 h at 180 °C under autogenic pressure. The product suspension was collected, centrifuged (Hettich, Universal 320, 20 min, 3000 rpm), washed with distilled water and ethanol and then dried at 60 °C for 24 h.

WS_2_ nanomaterials were obtained by a sulfurization of the WO_3_ nanowires. Sulfur powder (1.0 g, reagent grade, purified by sublimation) and WO_3_ nanowires (0.1 g) were placed in two different alumina boats at the center of a tubular reactor (quartz tube of 2″ diameter mounted in a 12″ single zone Thermolyne, split furnace). Before sulfurization, the reactor was evacuated to a base pressure of ~1 Torr and filled up with N_2_ at 1 bar. The evacuation and flushing steps were repeated three times to minimize O_2_ in the reactor. Sulfurization is performed for a period of 10 min at 500, 600, 700 or 800 °C in a flow of 400 sccm N_2_. Structural characterization. The crystal phase and microstructure of the obtained material is studied by the means of X-ray diffraction (Bruker D8 Discovery, Cu K_α_), micro-Raman spectroscopy (Horiba Jobin-Yvon Labram HR800 at λ = 488 nm), as well as scanning (FESEM, Zeiss Ultra Plus) and transmission electron microscopy (FEI Tecnai G2 20 X-Twin at 200 kV acceleration) techniques.

### Electrical and optical characterization

WS_2_ nanowire-nanoflake hybrid field-effect devices were fabricated on Si/SiO_2_ substrate (thermal oxide thickness 300 nm on p^+^-Si). The source and drain Au microelectrodes (300 nm thick Au sputtered on 45 nm Ti adhesion layer) were defined by photolithography. The WS_2_ nanowire and nanoflake hybrids were dispersed in acetone and deposited on the electrodes by drop casting. Temperature dependent current-voltage analysis was performed by probing the chips in a Linkam TMS 94 heating stage in air atmosphere using a computer-controlled Keithley 2636A source meter. The transfer characteristics were measured in a bottom-gated FET arrangement, while the optical properties were analyzed by illuminating the channel of the transistor devices using a standard collimated RGB light-emitting diode (emission centered at 623 nm, 517.5 nm and 466 nm with corresponding luminosity values of 800, 4000 and 900 mcd, respectively; chip-to-diode distance of 5 cm at an illumination angle of ~45°).

## Results and Discussion

**Figure 1:**
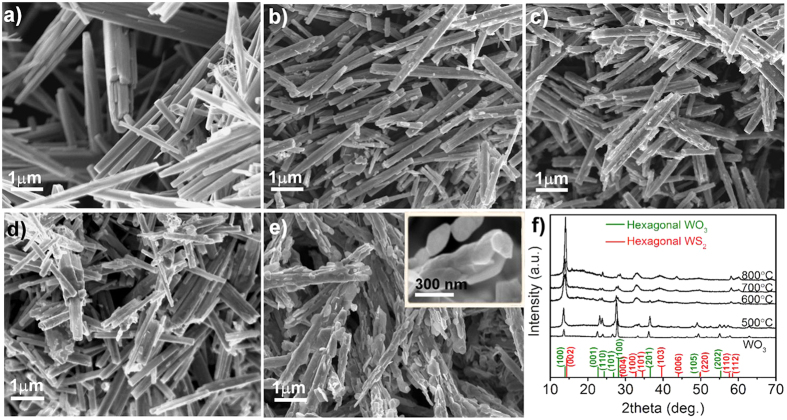
Structural change of WO_3_ during sulfurization. (**a**) Scanning electron micrograph of WO_3_ nanowires. Panels (**b–e**) display the corresponding products after sulfurization at 500 °C, 600 °C, 700 °C and 800 °C, respectively. (**f**) X-ray diffraction patterns of the corresponding products. Note: the reflection located around 24° corresponds to elemental sulfur on the surface of the WS_2_.

The cleaned product of the hydrothermally synthesized nanowires (Fig. 1a) shows a diameter distribution between 270 and 390 nm, while the length of the nanowires varies between 1 and 3 μm. Annealing of the nanowires in the vapor of sulfur does not induce considerable changes of the product morphology except for temperatures 700 °C or above when tiny flakes appear on the surface of the nanowires (Fig. 1b–e). The elemental composition obtained by energy-dispersive X-ray spectroscopy (EDS, Table S1) confirms that the product is transformed into WS_2_.The flakes are linked to the nanowires and look like being partially peeled off from the surface rather than subsequently attached to the nanowires. Although the growth mechanism for WO_3_ nanowires is now well understood and has been discussed in literature, the formation of WS_2_ nanowire-nanoflake hybrid is not yet clear. Shortly, one dimensional WO_3_ forms as follows: Initially a WO_3_ crystal nucleus is formed, which is then followed by WO_3_ primary prism particle formation due to the presence of Na^+^ and/or K^+^, which is present due to the formation of NaCl as byproduct^23^. Further crystal growth then happens along the [001] direction as it is energetically more preferential. The effect of NaCl and sulfate were studied in separate papers also, and both of them were found to serve as directing agent in the growth of one dimensional structures^24^,^25^. WS_2_ nanoflake formation however is less clear. It is believed that any minor mechanical stress caused by the transformation of oxide to sulfide might induce easy sliding and fragmentation of the layered WS_2_ sheets. As demonstrated earlier, similar but nanomechanical cleavage of MoS_2_ layers from a single crystal using an ultra-sharp W tip may be achieved^26^.

X-ray diffraction indicates the hydrothermally synthesized WO_3_ nanowires are of the WO_3_ hexagonal crystal phase [PDF 01-075-2187]. In the course of sulfurization however, the diffraction pattern changes particularly when the reaction temperature is increased suggesting the conversion of WO_3_ to WS_2_. Comparing the relative intensities of the most intensive reflections, it may be concluded that the WO_3_ phase dominates until 500 °C, while at 600 °C and above the hexagonal WS_2_ [PDF#841398] phase appears as the major constituent of the samples, with a complete oxide to sulfide transformation occurring at 800 °C. In the course of the reaction, oxygen in the anionic sites of the WO_3_ crystal is replaced by sulfur resulting in the formation of WS_3_, which then decomposes to WS_2_ as WS_3_ → WS_2_ + 1/8 S_8_^27^.

**Figure 2:**
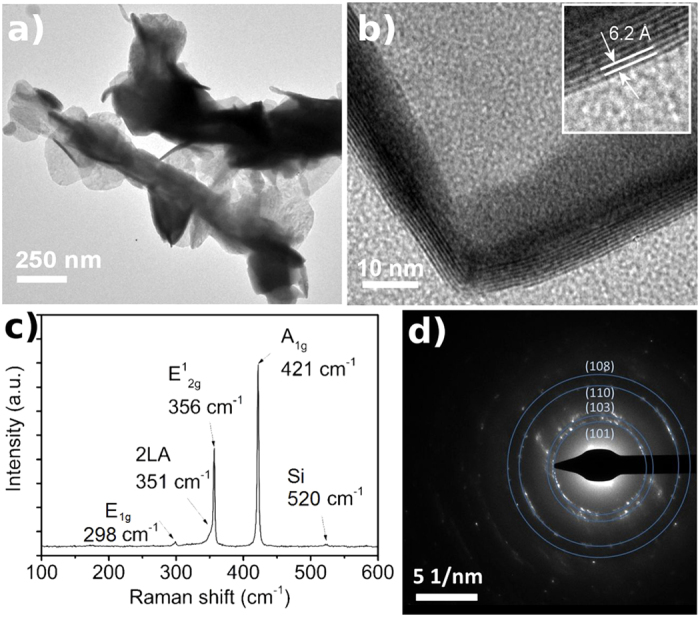
Structure of WS_2_ hybrid materials. (**a**) Low magnification transmission electron micrograph of WS_2_ nanowire-nanoflake hybrids synthesized at 800 °C. (**b**) High-resolution micrograph of a flake with layered crystal structure. Inset shows the d-spacing of the layers. (**c**) Raman spectrum of the corresponding WS_2_ nanowire/nanoflake hybrid material. (**d**) SAED pattern of WS_2_ nanowire-nanoflake hybrid synthesized at 800 °C.

Since complete transformation of the oxide to sulfide takes place only for the samples treated at 800 °C, the discussion will focus only on this product. High-resolution transmission electron microscopy was performed to observe the morphology and the layer separation in detail (Fig. 2a,b). The measured distance between the fringes in the lattice is approximately 0.62 nm Fig. 2(b), inset), which matches well the (002) d-spacing for the layered WS_2_ structure. Structures were also examined by SAED technique (Fig. 2d), where diffraction patterns revealed crystal structures corresponding to hexagonal WS_2_, which are in good agreement with XRD results. The layered flakes are partially wrapping the nanowires around, demonstrating a kind of exfoliation of the bulk structure. The typical thickness of the flakes is below 10 nm, i.e. the nanostructures consist of up to ~15 layers of WS_2_. XRD (Fig. 1f) and EDS (Table S1) techniques have been used to confirm the formation of the WS_2_ nanostructures. First, the sulfur powder turns into vapor carried by the nitrogen and deposited on the surface of the WO_3_ nanowire. The high temperature assists the reaction and conversion of the WO_3_ nanowire to WS_2_ nanowire/nanoflake (substitution of oxygen by sulfur).

Peaks in the micro-Raman spectrum of the sample synthesized at 800 °C (Fig. 2c) can be assigned to the in-plane E_2g_ mode (at ~352 cm^−1^) and to the out-of-plane A_1g_ mode (at ~420 cm^−1^) of crystalline WS_2_. As reported recently, the intensity ratios and peak frequencies of the WS_2_ Raman modes can give information about the number of layers^3^,^28^,^29^. In the case of multilayer (>5 layers) WS_2_ flakes though, the peak ratios of Raman spectra do not allow the exact determination of layer numbers, as the most prominent change in peak ratios happens at structures having 4 layers or less, as it was discussed by earlier papers. As a result of this, the determined intensity ratio of 0.5 of the peaks for the in-plane E_2g_^1^ and out-of-plane A_1g_ phonon modes can merely confirm that the prepared WS_2_ structure is indeed multilayer, which result is also in good agreement with our TEM observations.

**Figure 3:**
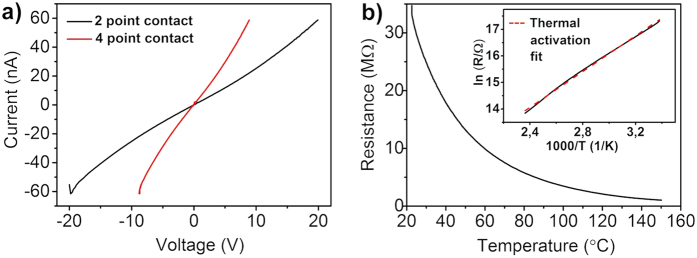
Electrical characteristics of WS_2_ hybrid films. (**a**) Current vs. voltage curves measured by 2 and 4-probe setups. (**b**) Temperature-dependent resistance and the corresponding Arrhenius plot for the thermally activated conduction.

Current-voltage curves measured by 2 and 4-probe setups display slightly nonlinear current-voltage characteristics (Fig. 3a). Since the 4-point analysis eliminates any barriers caused by the Au-WS_2_ interface, the contacts between the nanohybrids in the random network are also contributing to the contact barriers. Resistance versus temperature measurements (2-probe) carried out in air between 22 °C and 150 °C (Fig. 3b) show a decreasing resistance with temperature indicating a semiconducting behavior of WS_2_ nanohybrid films. The two commonly used models to describe the temperature dependence of the resistance of the semiconductors are the thermal-activation and variable range hopping models^12^. In the thermal-activation model, the temperature-dependent resistance can be written as *R* (*T*) = *R*_*0*_*·e*^(*E*/*kT*)^, where *E* is the activation energy and *k* is the Boltzmann constant. The linearized plot (i.e. ln (*R*) vs. *T*^−1^) gives an excellent fit for our dataset with a reasonable barrier of 0.29 eV obtained from the slope of the fitting parameter, thus thermally activated conduction is a plausible mechanism. Note, the variable range hopping model for 3-dimensional conduction (in which ln (*σ*) is linearly proportional to *T*^−1/4^, where *σ* is the conductivity)^30^,^31^ fits also very well (not displayed here) to our data. However the extracted values for localization length as well as density of electronic states are unphysical thus this latter model for the conduction mechanism may be ruled out.

**Figure 4:**
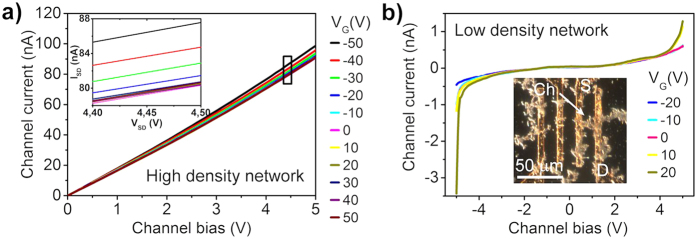
Output characteristics of the WS_2_ nanowire nanoflake hybrid based FET devices. Current-voltage curves for (**a**) high and (**b**) low density random networks of the hybrid nanowires between the source and drain electrodes. Inset in panel (a) displays a magnified plot for the outlined regime between 4.4 and 4.5 V for better visibility of the current values at different gate voltages. Inset in panel (b) shows a dark field optical micrograph of a device.

Although the properties of more common chalcogenides have already been extensively investigated^32^,^33^,^34^, WS_2_ nanohybrid materials to or knowledge have not been studied yet. In order to investigate the electrical characteristics of the nanohybrid networks, bottom-gated field-effect devices are prepared on a Si chip of both high and lower synthesis temperature materials. By sweeping the source-drain voltage (V_ds_) between the Au electrodes the current is measured in the WS_2_ channel, while a gate voltage (V_g_) through the conductive Si/Al/Au substrate is applied. Materials prepared at lower temperatures (500 and 600 °C) exhibited no field effect behavior (Fig. S1), while films made of samples prepared at 800 °C showed a rather weak but measurable field effect. This may be attributed to trapped carriers at the contacts of the nanohybrid particles in the films and also at the interface of the particles and substrate, thus reducing carrier mobility^35^. The field-effect mobility estimated from the conventional two-probe expression^36^ 
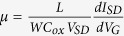
 is about ~0.02 cm^2^V^−1^s^−1^ (where *L* and W are the channel length and width, 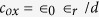 is the gate capacitance (*d* = 300 nm and *ε*_*r*_ = 3.9) and *dI*_*SD*_/*dV*_*G*_ is the transconductance (~2.2 × 10^−10^ A/V) estimated from the *I–V* slopes measured at different gate voltages (Fig. 4a).

The measured value is comparable with results found for MoS_2_ and WS_2_ based FETs applying sulfurized metal films (0.004–0.04 cm^2^V^−1^s^−1^)^37^,^38^ and CVD grown MoS_2_ from MoO_3_ and S precursors (0.02 cm^2^V^−1^s^−1^)^39^ or CVD grown WS_2_ from WO_3_ and S precursors (0.01 cm^2^V^−1^s^−1^)^40^. Some other types of WS_2_ materials exhibit higher carrier mobilities due to the smaller number of crystal interconnections as opposed to the smaller nanowire materials where charge needs to pass through a higher number of junctions. Such examples are single crystal WS_2_ with d ≈ 20 μm crystal size (100 cm^2^V^−1^s^−1^)^41^, WS_2_ films (9–15 cm^2^V^−1^s^−1^)^42^, exfoliated single layer WS_2_ (80 cm^2^V^−1^s^−1^)^43^ or WS_2_ nanotubes (50 cm^2^V^−1^s^−1^)^44^. Taking this into consideration, we speculate that the mobility may be increased by improving the semiconductor-metal contact as well as by using individual (or only a few) WS_2_ nanoparticles instead of thick high density random networks between the electrodes, which will result in a smaller number of crystal interconnections between the electrodes.

To verify this assumption, transistors having only a few nanowire-nanoflake hybrid particles connecting the source and drain electrodes were fabricated. The corresponding current-voltage characteristics as shown in Fig. 4(b) are highly nonlinear and have pronounced gate effect owing to the lesser amount of nanohybrid to nanohybrid contacts in the transistor channel. Unlike in the high-density networks, here the channel has a negative character, as it opens at positive gate voltages. The estimated apparent carrier mobility (0.18 cm^2^V^−1^s^−1^) of WS_2_ in the channel is at least an order of magnitude higher than in the devices with high-density WS_2_ nanohybrid networks supporting our hypothesis regarding the performance-limiting effect of excessive numbers of contacts in the films.

**Figure 5:**
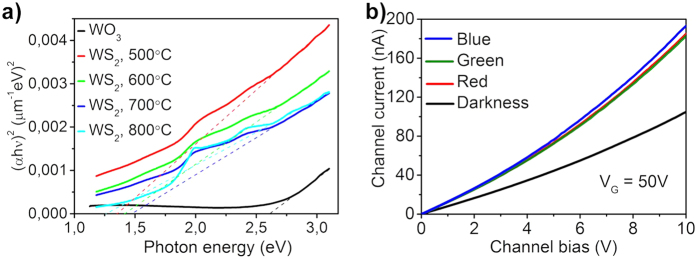
Optical properties of WS_2_ nanohybrids. (**a**) Tauc plot for direct band-to-band transition derived from total reflectance measurements on the original and sulfurized powders. Dashed lines represent fitting of the linear sections of each curve. Intersections of the dashed lines with the horizontal axis define the band gap. (**b**) Current-voltage characteristics of a high density FET under different LED illumination conditions (red, green and blue centered at 623 nm, 517.5 nm and 466 nm, respectively).

Optical measurements of powder samples were carried out to analyze further the properties of the samples. By using a spectrophotometer system (Optronic Laboratories, USA) equipped with an integrating sphere, we measured total reflectance within the 400–1100 nm spectral range from the scattering powders mounted on a microscope slide with double-sided Scotch tape. Then we calculated the optical absorption, from which the band gap of the materials synthesized at various conditions was derived. From the Tauc plot for direct interband transitions (Fig. 5a) we found that the sulfurization results in a clear decrease of the band gap from ~2.6 eV to ~1.4 eV due to the chemical transformation of tungsten oxide to sulfide. The obtained values are in good agreement with literature values of the band gap for WO_3_ after sulfurization^45^.

The photoelectric response of high density network FET devices is studied by illuminating the chips with light-emitting diodes (LEDs) of different color. As displayed in Fig. 5(b), the channel current is found to be increased under illumination using any of the LEDs. However, the effect is somewhat more pronounced when exposing the channel to photons of shorter wavelength due to the better optical absorption and more efficient photogeneration of carriers in the semiconductor. Interestingly, the chip illuminated with the green LED, which is ~4-times brighter than the blue or red source does not cause any extraordinary change in the channel current compared to the other two LEDs. This indicates again that the large contact resistance between the nanowires limits the current and adversely affects the device performance. Anyhow, the magnitude of change in the channel conductance is comparable with those measured for individual single-layer nanoflakes^46^.

## Conclusions

We have synthesized layered semiconducting WS_2_ nanowire-nanoflake hybrid materials by a simple sulfurization of WO_3_ nanowires at elevated temperatures. Field-effect transistors are demonstrated using bottom gated chips with channel made of random networks of the hybrid material drop casted between source-drain electrodes. The same structures were also applied as light detectors with good response to visible photons. The performance of both FET and photodetector devices is limited by the large contact resistance between the hybrid nanomaterials in the random network, which may be overcome by using optimized device structures having only a few nanowire-nanoflake hybrid particles in the channel. The work presented here suggests that nanowire-nanoflake hybrids of WS_2_, which can be synthesized by using simple chemical methods in large quantities, are competitive counterparts of few layer chalcogenides in electrical and optoelectronic applications.

## Additional Information

**How to cite this article**: Alene, G. *et al*. A novel WS_2_ nanowire-nanoflake hybrid material synthesized from WO_3_ nanowires in sulfur vapor. *Sci. Rep.* **6**, 25610; doi: 10.1038/srep25610 (2016).

## Supplementary Material

Supplementary Information
