# Supplementary material for: A novel WS2 nanowire-nanoflake hybrid material synthesized from WO3 nanowires in sulfur vapor
Source: Sci Rep. 2016 May 16;6:25610. doi: 10.1038/srep25610 (PMC4867582; doi:10.1038/srep25610)
Supplement: Supplementary Information [file srep25610-s1.pdf]

Supplementary information for "A novel WS_2 nanowire-nanoflake hybrid material synthesized from WO_3 nanowires in sulfur vapor"

Georgies Alene,[1] Aron Dombovari,[1] Teemu Sipola,[1] Robert Puskás,[2] Akos Kukovecz,[2,3] Zoltán Kónya,[2,4] Alexey Popov,[5] Jhih-Fong Lin,[1] Gabriela Lorite,[1] Melinda Mohl,[1] Geza Toth,[1] Anita Lloyd-Spetz,[1,6] Krisztian Kordas[1]

[1] *Microelectronics and Materials Physics Laboratories, Department of Electrical Engineering, University of Oulu, P.O. Box 4500, FI-90014 Oulu, Finland*

[2] *Department of Applied and Environmental Chemistry, University of Szeged, Rerrich Bela ter 1, H-6720 Szeged, Hungary*

[3] *MTA-SZTE "Lendület" Porous Nanocomposites Research Group, Rerrich Bela ter 1, H-6720 Szeged, Hungary*

[4] *MTA-SZTE Reaction Kinetics and Surface Chemistry Research Group, Rerrich Bela ter 1 H-6720 Szeged, Hungary*

[5] *Optoelectronics and Measurement Techniques Laboratory, Department of Electrical Engineering, University of Oulu, P.O. Box 4500, FI-90014 Oulu, Finland*

[6] *Department of Physics, Chemistry and Biology, Linköping University, SE-58183 Linköping, Sweden*

Table S1: Content of elements in different samples measured by EDS. All numbers are given in wt% values.

Sample	Average oxygen content	Average sulfur content	Average tungsten content
500 °C 10 min	15.1	12.6	72.3
500 °C 60 min	10.4	11.0	78.6
600 °C 10 min	7.6	12.6	79.8
600 °C 60 min	6.7	16.5	76.8
800 °C 10 min	7.5	24.4	68.1

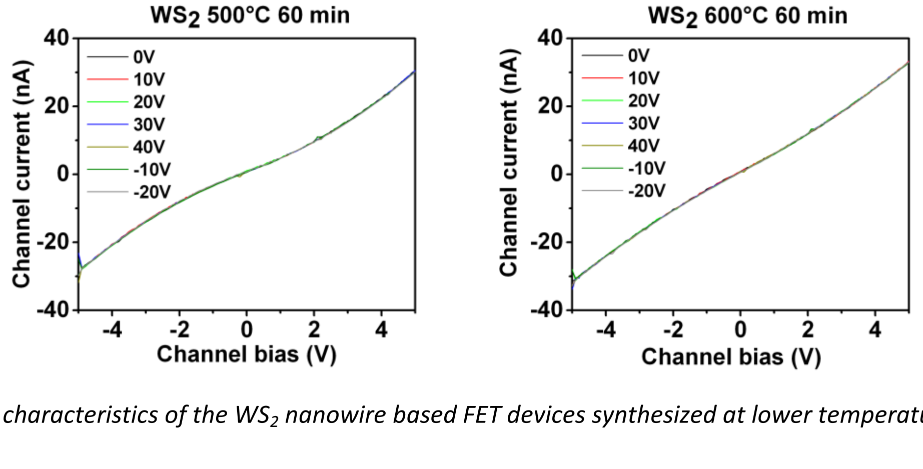

Figure S1: Output characteristics of the WS_2 nanowire based FET devices synthesized at lower temperatures.
